# Incidence of primary congenital hypothyroidism over 24 years in Finland

**DOI:** 10.1038/s41390-022-02118-4

**Published:** 2022-06-03

**Authors:** Emmi Danner, Laura Niuro, Hanna Huopio, Harri Niinikoski, Liisa Viikari, Jukka Kero, Jarmo Jääskeläinen

**Affiliations:** 1 Department of Pediatrics, University of Eastern Finland and Kuopio University Hospital, Kuopio, Finland; 2 Department of Pediatrics, University of Turku and Turku University Hospital, Turku, Finland

## Abstract

**Background:**

A rise in the incidence of congenital hypothyroidism (CH) has been reported worldwide. This nationwide study aimed to describe the secular trends and current incidence of CH in Finland.

**Methods:**

Two independent study cohorts, a national and a regional, were collected from national registers and patient records. The national cohort represents all CH cases born in Finland between 1994 and 2017. Birth data, results of the screening test, and the incidence of CH were reviewed.

**Results:**

Between 1994 and 2017, 1,400,028 children were born in Finland. Of these children, 503 were diagnosed with primary CH (incidence 1:2783). Male-to-female sex ratio was 1:2.0. The nationwide incidence was 33 cases per 100,000 live births between 1994 and 1999, 38 cases per 100,000 live births between 2000 and 2005, 40 cases per 100,000 live births between 2006 and 2011, and 33 cases per 100,000 live births between 2012 and 2017. In the regional cohort (*n* = 139), the incidence of transient CH was 3.6%. The incidence of mild, moderate, and severe CH remained constant.

**Conclusions:**

In Finland, the incidence of CH has not changed during the 24-year study period.

**Impact:**

- As opposed to recent reports worldwide, the incidence of congenital hypothyroidism has not changed between 1994 and 2017 in Finland.
- The proportions of mild, moderate, and severe congenital hypothyroidism did not change significantly over the study period.
- Lowering the TSH cut-off limit or increasing immigration did not affect the incidence rate of primary congenital hypothyroidism in Finland.

## Introduction

Congenital hypothyroidism (CH) is the most common congenital endocrine disease. Thyroid hormone has a crucial effect on several organ systems. If CH is not detected and treated effectively and promptly after birth, it may severely impair neurocognitive development and growth.^1^ Therefore, newborn screening programs for primary CH were introduced in many Western countries approximately four decades ago.

A rise in the incidence of CH has been reported worldwide and is attributed to various contributing factors. During the past decades, thyrotropin (thyroid-stimulating hormone (TSH)) cut-off values have been lowered in many countries, which affects the incidence rate of less severe CH,^2–5^ but also the number of more severe cases has increased.^6^ In some countries, the increasing number of preterm babies has been postulated to explain a part of the increased incidence of CH.^4,7,8^ Furthermore, some studies suggest that the incidence of CH has increased due to changes in ethnic composition.^8–10^ Still, in some studies the reason for increasing incidence has remained unknown.^11^

In Finland, nationwide cord blood screening for CH with complete coverage was implemented in 1980.^12^ In the early 80s, the incidence of CH was 1:2637^12^ and between 1985 and 1990 1:3969.^13^ However, the national incidence has not been studied since. This nationwide study was launched to determine the secular trends and current incidence of CH in Finland.

## Methods

We collected two independent study cohorts, a national and a regional.

For the nationwide study, the study population was identified from four national registers: the Prescription Register maintained by the Social Insurance Institution of Finland, the Care Register of Health Care (earlier the Hospital Discharge Register), the Medical Birth Register, and the Register of Congenital Malformations maintained by The Finnish Institute for Health and Welfare. These registers are linkable through unique personal identity codes that are assigned for all residents. We used data from January 1, 1994 to December 31, 2017. The data were anonymized in Statistics Finland and provided to the researchers’ use by a remote access system.

The inclusion criteria were the diagnosis of CH (ICD10 E03.1 or E03.0/ICD9 243) under the age of 2 years (the Care Register of Health Care) and/or the purchase of the levothyroxine under the age of 1 year (the Prescription Register) and/or the diagnosis of CH in the Medical Birth Register and/or in the Register of Congenital Malformations. The exclusion criteria were (a) diagnosis of CH registered for less than 2 months (expect in case of death under the age of 1 year) or (b) diagnosis of end-stage renal disease (ICD10 N18/ICD9 585 or 586) or panhypopituitarism (ICD10 E23.00/ICD9 253.2). This data represents all the CH cases born in Finland between 1994 and 2017 (*n* = 503).

A matched control cohort was created using the Medical Birth Register. With two exceptions who received only 1 control, 2 controls for each patient were randomly selected. The control group was matched for date of birth (+/−3 months), sex, mother’s age at birth (+/−2 years), number of fetuses, parity (1, 2, 3, or more), and place of birth (hospital district).

Birth data from the patients and controls and the yearly birth rate were collected from the Medical Birth Register.

A regional cohort was collected to get more detailed information (laboratory values at birth and at diagnosis, imaging of thyroid, and results of re-evaluation) from CH patients. For the regional study, data were collected from the catchment areas of Kuopio and Turku university hospitals. Patients were identified by a diagnosis-based search from the patient records from 1994 to 2017. The inclusion criteria were abnormal screening and confirmatory test results (*n* = 131). In borderline cases, patients were included in the study when a pediatric endocrinologist or a pediatrician-diagnosed primary CH and levothyroxine was started before the age of 14 days (*n* = 8). The fulfilment of the inclusion criteria was manually verified from the medical records by two of the authors (E.D., L.N.). This study cohort represents all the CH cases born in Turku and Kuopio university hospitals’ catchment areas between 1994 and 2017 (*n* = 139).

In 1980s in Finland, laboratory testing for CH was centralized. From the beginning of 1990s, the CH screening program has been multicentric and is managed by central hospitals and regional laboratories. After regionalizing the screening program, laboratory methods have varied between laboratories and over time. The cut-off limits for both initial and confirmatory screening have remained unchanged or lowered depending on the method used in each laboratory. The cut-off limit has varied between TSH 25 and 40 mU/l for the initial screening and between TSH 20 and 40 mU/l for the confirmatory screening. During the study period, the same screening strategy has been used for both full-term and preterm babies.

The screening test for primary CH is umbilical cord blood TSH (mU/l). Cord serum samples are collected in tubes for analysis. Coverage being near to 100%, practically all the children born in Finland are screened.^12,14^ Infants with TSH below the threshold are considered as free of CH. In a recent Finnish study, 0.76% of cord blood TSH results were false positive.^15^ If the TSH value is over the threshold, a confirmatory test (TSH and free thyroxine (fT4)) is performed after 72 h of age. In this study, the median age for the confirmatory test was 74 h (interquartile range (IQR) 72–84 h). The diagnosis is set, and the follow-up is carried out by a pediatric endocrinologist or a pediatrician in a central hospital. Re-evaluation of the diagnosis, if needed, is performed at 2 or 3 years of age to detect transient CH.

Imaging of the thyroid (scintigraphy) was routinely performed in the 1980s. However, as routine imaging practice did not provide any additional information guiding the treatment or follow-up of CH, it was later rejected. Thereafter, ultrasound or scintigraphy has been performed only for selected cases, e.g., when assessing necessity of re-evaluating the diagnosis or when the required levothyroxine dosage is abnormally low.

In the regional study, we assessed the severity of CH by using both initial TSH concentration at birth and fT4 concentration in the confirmatory test. According to TSH, we divided the infants with primary CH into three different groups: severe = TSH > 100 mU/l (*n* = 103), moderate = TSH 50–100 mU/l (*n* = 21), and mild = TSH < 50 mU/l (*n* = 11). According to fT4, patients were divided into two groups: severe = fT4 < 5 pmol/l (*n* = 35), and mild = fT4 ≥ 5 pmol/l (*n* = 90). There were four missing TSH values and 14 missing fT4 values. If TSH of fT4 values were outside the range of detection, we used the current laboratory-specific lowest or highest limit of detection, appropriately, as the result for any calculations.

Birth length, birth weight, and head circumference standard deviation score were calculated by the latest Finnish growth references.^16^

The data were analyzed using the Statistical Package for Social Sciences (SPSS for Windows, Version 27, IBM Corp., Armonk, NY). Due to the non-normal distribution of data, statistical analyses were presented as medians and IQRs (25th and 75th percentiles). Mann–Whitney *U*-test was used for comparisons between cases and controls in continuous variables. For non-continuous variables, Pearson Chi-square test was used. The independent samples Kruskal–Wallis test was used for group comparisons. A Poisson regression model was used to characterize trends in the incidence of mild and severe CH over the timespan of the study. The model was adjusted to take into account variations in birth rate. A *p* value <0.05 was considered statistically significant.

The Finnish Institute for Health and Welfare, the Social Insurance Institution of Finland, and Statistics Finland granted permission for this study. The study was approved by the Ethics Committee of the Northern Savo Hospital District.

## Results

Between 1994 and 2017, 1,400,028 children were born in Finland. Of these children, 503 (333 girls) were diagnosed with primary CH (incidence 1:2783). Female-to-male sex ratio was 2.0. The birth data of the cases compared to their controls are represented in Table 1. The neonates with CH had 0.5 cm (0.4 SD) larger head circumference at birth compared to their controls (*p* < 0.001) They were born 5 days past due date (median), whereas the babies in the control group were born on due date (*p* < 0.001).

**Table 1:** Birth data of all patients diagnosed with CH in Finland between 1994 and 2017 compared to their controls.

	Cases *n* = 503	Controls *n* = 1004	*p*
Birth weight (g), median (IQR)	3620 (3211 to 3975)	3510 (3166 to 3855)	0.148^b^
Birth weight (SDS), median (IQR)	−0.1 (−0.9 to 0.6)	−0.1 (−0.8 to 0.5)	0.736^b^
Birth length (cm), median (IQR)	50.0 (49.0 to 52.0)	50.0 (49.0 to 51.0)	0.750^b^
Birth length (SDS), median (IQR)	−0.2 (−0.8 to 0.4)	−0.1 (−0.8 to 0.6)	0.057^b^
Head circumference at birth (cm), median (IQR)^a^	35.5 (34.5 to 36.5)	35.0 (34.0 to 36.0)	**<0.001**^b^
Head circumference at birth (SDS), median (IQR)^a^	0.3 (−0.5 to 1.1)	−0.1 (−0.8 to 0.7)	**<0.001**^b^
Small for gestational age (birth weight <2.0 SDS, % of cases)	5.4%	3.6%	0.104^c^
Large for gestational age (birth weight >2.0 SDS, % of cases)	2.8%	2.8%	0.993^c^
Duration of pregnancy (days from due date), median (IQR)	+ 5 (−5 to 12)	0 (−7 to 6)	**<0.001**^b^
Prematurity <37 weeks (% of cases)	7.0%	5.1%	0.138^c^
Male-to-female sex ratio	1:2.0	NA	
Mother’s age at birth, median (IQR)	30 (26 to 34)	NA	
Multiple pregnancy (% of cases)	3.2%	NA	
Persons with foreign background (% of cases)	5.0%	4.4%	0.607^c^

*IQR* interquartile range, 25th and 75th percentiles, *NA* not applicable, matched controls.

Bold values represent statistical significance *p* < 0.05.

^a^Data available from 2004.

^b^Mann–Whitney *U*-test.

^c^Pearson Chi-square.

**Fig. 1:**
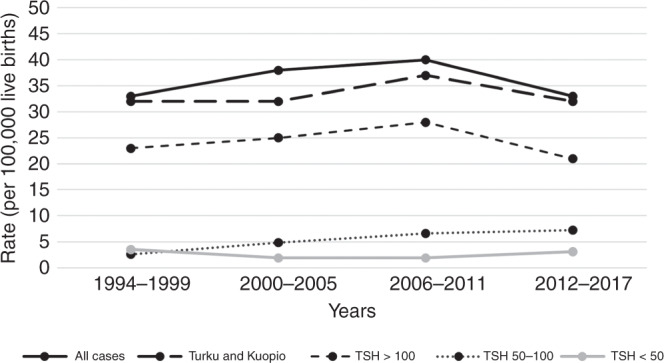
The incidence of primary congenital hypothyroidism (CH). The incidence of CH in Finland and in Kuopio and Turku university hospitals’ catchment areas over 24 years (1994–2017) per 100,000 live births according to initial TSH concentration from umbilical cord.

During the same period in Kuopio and Turku university hospitals’ catchment areas, 421,246 children were born and 139 of them (83 girls) were diagnosed with primary CH (incidence 1:3053). Five of them had transient CH (3.6%). The incidence of CH did not change over the study period (Fig. 1). The nationwide incidence was 1:3050 between 1994 and 1999, 1:2619 between 2000 and 2005, 1:2519 between 2006 and 2011, and 1:3031 between 2012 and 2017. During the same study period, the incidence of CH was similar in the regional cohort varying between 1:2719 and 1:3173.

Over the study period, there were no statistically significant changes in TSH levels in the screening test nor in the confirmatory test (Table 2). Most of the cases had a TSH level of over 100 mU/l both in the initial (76%) and in confirmatory (75%) tests.

**Table 2:** Diagnostic features according to year of birth in all patients born in Kuopio and Turku University Hospitals’ specific catchment areas between 1994 and 2017.

	1994–1999 *n* = 36	2000–2005 *n* = 33	2006–2011 *n* = 39	2012–2017 *n* = 31	*p*^a^
Umbilical cord blood TSH, mU/l, median (IQR)	347 (130–455)	325 (160–420)	240 (109–310)	260 (60.0–470)	0.231
Plasma TSH levels at diagnosis, median, mU/l (IQR)	358 (95.2–498)	290 (101–411)	100 (83.4–326)	226 (93.0–278)	0.086
Plasma fT4 levels at diagnosis, median, pmol/l (IQR)	8.7 (4.5–12.7)	5.7 (4.2–12.3)	11.2 (5.6–17.8)	10.0 (6.0–18.2)	0.078

*IQR* interquartile range, 25th and 75th percentiles.

^a^Independent samples Kruskal–Wallis test.

**Fig. 2:**
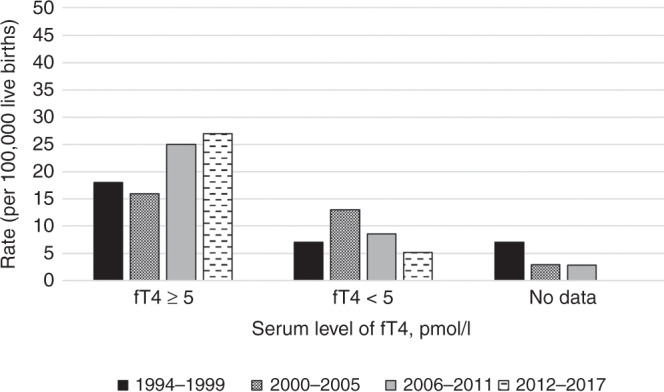
The incidence of primary congenital hypothyroidism (CH) by the severity of the disease. The incidence of CH in Kuopio and Turku university hospitals’ catchment areas from 1994 to 2017 per 100,000 live births according to initial plasma fT4 concentration at diagnosis.

The incidence of mild, moderate, and severe CH remained constant over the study period (Fig. 1). However, an increasing trend in the incidence of mild CH (*p* = 0.075) and a decreasing trend in severe CH (*p* = 0.492) was detected when the severity of the disease was defined by fT4 concentration in the confirmatory test (mild = fT4 ≥ 5 pmol/l, severe <5 pmol/l) (Fig. 2).

## Discussion

Our nationwide study showed a stable incidence of CH in Finland during the 24-year period from 1994 to 2017.

As opposed to our findings, an increase in the incidence has been described in many studies worldwide.^17^ The most likely explanation for the increased incidence has been the progressive lowering of the diagnostic TSH cut-off value and re-screening strategy for neonates considered being in a higher risk for CH, such as twins and premature newborns.^4^ In Italy, the decrease of the cut-off values has doubled the increase of CH from 1:2200–4000 to 1:1090.^3,7^ Lowering the cut-off limits has led to detect not only additional cases of transient but also permanent CH.^2,3,18,19^ In some studies, also the incidence of more severe forms of CH has increased.^6^

In Finland, the cut-off limit of the screening test is among the highest in Europe,^14^ mainly due to the use of umbilical cord blood sample instead of dried blood spot testing used in most of Western countries. The confirmatory test is performed using venous or capillary samples. The serum TSH cut-off limit 20–40 mU/l equates approximately 12–25 mU/l on filter paper.^9,20^ During the study period, the cut-off limits have remained unchanged or lowered depending on the method used in each laboratory.

In some studies, an increasing number of preterm births and the improved survival rate of preterm and low birth weight infants have been suggested as one of the reasons behind the increasing incidence of CH.^7,8^ In Finland, the rate of preterm births has not increased. In 1995, 5.1% of all births were preterm and in 2017 5.3%.^21^ The survival rate of very preterm babies has increased between 1995 and 2017 from 80 to over 90%.^22^ Still, these changes did not affect the rate of detected CH in Finland. However, re-screening of twins and preterm babies has not been universally performed in Finland.

One suggested factor related to the increasing incidence of CH is a change in demographic characteristics.^8–10^ In a study from New Zealand, an increase in the incidence of CH was observed, but there was no change in ethnic-specific incidences,^9^ reflecting the role of immigration as an explanatory factor for the increasing CH incidence. This theory is supported by a higher CH incidence especially in Asians as compared to other ethnicities.^23,24^ In the US, the ethnic-specific incidence has increased specially among Hispanic newborns.^8^ On the other hand, genetic diversity in blacks may be a protective factor against the dysgenetic form of CH, whereas decreased genetic diversity and greater deleterious genetic variation in Caucasians set them at higher risk for developing thyroid dysgenesis.^10^

In Finland, the population with foreign background (born abroad or both parents born abroad) was fivefold larger in 2017 than in 1994 (384,123 and 73,463 persons, respectively).^25^ Immigration flow from Asia represents a minority of foreigners in Finland. Most of the persons with foreign background come originally from the countries of former Soviet Union,^25^ which may explain that immigration has not affected the incidence rate of CH in Finland.

Changes in iodine intake can also affect the incidence of CH. In Finland, the dietary iodine intake has decreased during the study period and is relatively low compared to other Western countries.^26^ However, it is still considered as adequate or almost adequate according to World Health Organization’s guidelines.^27^

In the literature, the incidence of transient CH varies between 5 and 50% of cases diagnosed with CH. In a Korean study,^28^ 47% of cases diagnosed with CH were transient. From the infants in Italy diagnosed with CH, 30–35% had transient CH.^7^ In Greece, the overall percentage of transient CH was 11.2% of all CH cases and higher among premature compared with full-term infants (25.8 and 7.5%, respectively).^19^ In North America, the incidence of transient CH was 5–10% in children positive for CH in mass screening.^29^

Our nationwide cohort did not allow a separation between transient and permanent CH. In the regional cohort, the incidence of transient CH was 3.6% of all infants diagnosed with CH, which is less than usually reported in the literature. A relatively high cut-off limit of the screening test may reduce the number of transient CH cases detected in Finland.

A female preponderance of CH has been earlier described.^24^ In the US national data set, the female-to-male ratio for CH was 1.56 instead of the previously reported ratio of 2.0.^24^ In Texas, the incidence rate of CH increased among male newborns causing the change in female-to-male ratio from 2.0 to 1.5.^8^ In Finland, the female-to-male ratio in the national study was 2.0 as expected.

A U-shaped association between the prevalence of CH and birth weight^24^ and also between the prevalence of CH and gestational age^30^ has been earlier described. Similarly to the current study, a clear tendency toward prolonged pregnancy has been observed also in previous Finnish studies.^31^ In addition, as in the current study, neonates with CH have been reported to have large head circumference at birth.^32,33^

There were some limitations in this study. The incidence is counted from all live born children, not from the children screened for CH. During the study period, only 0.1‰ of all children were born at home. Thus, the coverage of screening test in hospital births is practically 100%. Another limitation is the variation of the TSH detection assays used in different laboratories over time. Moreover, due to restrictions of the national registers, it is possible that the nationwide cohort includes some screening negative cases. On the other hand, the strength of this study was the parallel analysis of two cohort populations, nationwide and a more detailed regional. A similar incidence of CH was observed in both cohorts verifying their representativeness and strengthening the quality of our data.

In conclusion, there were no changes in incidence of CH during the 24-year study period in Finland.

## Data Availability

The datasets generated and analyzed during the current study are not available due to data privacy and ethical and legal concerns.
